# Neurotransmitter 5-HT Further Promotes LL-37-Induced Rosacea-like Inflammation Through HTR3A

**DOI:** 10.3390/ijms26073156

**Published:** 2025-03-28

**Authors:** Haojie Ma, Jing Liu, Fengfeng Chen, Yonghua Zhou, Cheng Yang, Bingtian Zhao

**Affiliations:** 1 Key Laboratory of Synthetic and Biological Colloids, Ministry of Education, School of Chemical and Material Engineering, Jiangnan University, Wuxi 214122, China; mhj19825305446@163.com (H.M.); lj15735649029@163.com (J.L.); cff@jiangnan.edu.cn (F.C.); 2 Key Laboratory of National Health Commission on Parasitic Disease Control and Prevention, Jiangsu Provincial Key Laboratory on Parasite and Vector Control, Jiangsu Institute of Parasitic Disease and Public Health Research Center of Jiangnan University, Wuxi 214064, China; toxo2001@163.com

**Keywords:** rosacea, inflammation, 5-HT, macrophages, HTR3A

## Abstract

Rosacea is a chronic inflammatory skin disease and is usually accompanied by extensive macrophage infiltration. There is growing evidence suggesting that neurotransmitter 5-hydroxytryptamine (5-HT) plays a crucial role in inflammatory reactions. However, the interaction between 5-HT and rosacea is still unclear. Here, we hypothesized that the inflammation of rosacea is partly caused by 5-HT, and we investigated the underlying mechanism. In this study, we employed a rosacea model induced by LL-37, which is usually applicated as a rosacea stimulator, to investigate the effects of 5-HT on rosacea in vitro and in vivo. In LL-37-(4 μM)-induced THP-1-derived macrophages, 5-HT (400 μM) further promoted the secretion of inflammatory cytokines and polarized macrophages towards M1 phenotype, which could promote an inflammatory response. Further research revealed that exposure to LL-37 and 5-HT (L5) selectively upregulated *HTR3A* mRNA expression but not *HTR2A* or *HTR7* and induced colocalization of 5-HT with HTR3A. Notably, application of antagonist tropisetron (TPS) and siRNA of *HTR3A* suppressed L5-induced inflammation. Meanwhile, 5-HT (25 μg each injection a total of three times) deteriorated skin erythema, stimulated dermal inflammatory cell infiltration, and promoted the secretion of inflammatory cytokines in LL-37 (40 μL and 320 μM each injection a total of four times) induced rosacea-like mice, while these undesirable effects were reversed by using TPS. Our findings revealed that neurotransmitter 5-HT further promoted LL-37-induced rosacea-like inflammation through HTR3A. Our study highlights HTR3A as a promising therapeutic target, which warrants further in-depth investigation into its clinical applicability.

## 1. Introduction

Rosacea is a chronic inflammatory skin disease with an estimated global prevalence of 5.46% [1]. Rosacea directly diminishes the quality of life and is associated with significant psychosocial issues, including depression and inferiority [2]. Clinically, rosacea is classified into four subtypes, namely erythema, telangiectatic, papulopustular, and ocular rosacea [3]. In contrast to other inflammatory skin diseases, such as psoriasis and atopic dermatitis, neurogenic inflammation plays a pivotal role in the pathogenesis of rosacea [4,5,6]. Recent research uncovered that trigger factors, like heat and spicy food, are capable of directly activating cutaneous sensory neurons, resulting in immune dysregulation and subsequent manifestation of rosacea lesions [7]. This process may be mediated by neurogenic inflammation—a phenomenon in which stimulation of sensory nerve endings triggers the release of neurotransmitters. Notably, the clinical features of neurogenic inflammation closely resemble the hallmark symptoms of rosacea, suggesting a potential mechanistic link between neuronal activation and cutaneous immune dysregulation in this disease [4]. However, the mechanism of neurogenic inflammation in rosacea is unclear, and there is a lack of clinical treatments for neuroimmune imbalances in rosacea.

5-HT, which is widely regarded as a neurotransmitter, functions not only in the nervous system but also in the peripheral immune system, including the skin [8]. Nearly 90% of 5-HT is synthesized within the intestine and subsequently transported via platelets to diverse parts of the body to participate in various physiological regulations including immunoregulation [9]. In the skin, 5-HT can be synthesized by keratinocytes, melanocytes, and immunocytes in addition to being obtained from platelets [10]. At the inflammatory sites, 5-HT plays a pivotal role in modulating the immune response, particularly through the recruitment of immunocytes [11]. Notably, dysregulation of 5-HT signaling has been implicated in various inflammatory skin diseases. For instance, Kim et al. demonstrated that acupuncture may alleviate atopic dermatitis-like skin inflammation by selectively blocking 5-HT2 and 5-HT7 receptors [12]. Meanwhile Younes et al. demonstrated that the number of 5-HT-positive cells is significantly higher in patients with inflammation compared to normal skin, highlighting the therapeutic potential of targeting 5-HT pathways in inflammatory conditions [13]. Additionally, 5-HT requires binding to its receptors (HTRs) to exert biological functions [8]. It is also crucial to identify the key receptor subtypes involved in 5-HT-driven inflammation. However, the interaction between 5-HT and rosacea is still unclear. It is urgent to investigate the 5-HT signaling conducted in rosacea.

The pathological characteristics of rosacea are closely related to the inflammatory microenvironment driven by macrophages [14]. Meanwhile, macrophages highly express a variety of 5-HT receptors, which can directly respond to signals of 5-HT released by sensory neurons, forming a nerve immune two-way communication [15,16]. This property causes macrophages to become the key target cells to analyze the mechanism of 5-HT in rosacea.

Building upon evidence implicating 5-HT in neuro-immune dysregulation, we hypothesized that 5-HT would exacerbate rosacea by amplifying inflammatory responses. Therefore, in this study, series of experiments were conducted to investigate the role of 5-HT in rosacea-associated inflammation. By integrating neuronal signaling with cutaneous immunology, this work provides novel insights into neurogenic inflammation in rosacea, potentially informing targeted therapies for rosacea.

## 2. Results

### 2.1. Effects of 5-HT and LL-37 on Inflammatory Cytokines and Phenotypes of Macrophages

**Figure 1:**
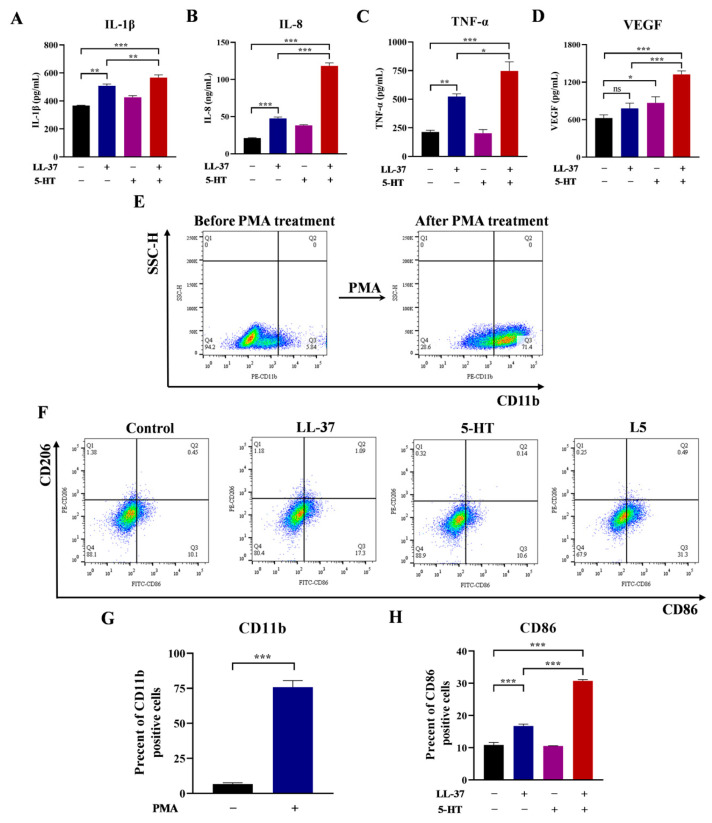
Effects of 5-HT and LL-37 on inflammatory cytokines and phenotypes of macrophages. (**A**–**D**) After incubation with LL-37 (4 μM) or 5-HT (400 μM) for 24 h, the ELISA results of the expressions of IL-1β, IL-8, TNF-α, and VEGF of control (black), LL-37 (blue), 5-HT (purple), and L5 (red) treated cell groups. (**E**) Representative flow cytometry detection of CD11b-positive cells after treatment with PMA. (**F**) Representative flow cytometry detection of CD86- and CD206-positive cells after incubation with LL-37 or 5-HT for 24 h. (**G**) The percent of CD11b-positive cells of control (black) and PMA (blue) treated groups. (**H**) The percent of CD86-positive cells of control (black), LL-37 (blue), 5-HT (purple), and L5 (red) treated groups. Data represent mean ± SEM for three independent experiments. *, *p* < 0.05; **, *p* < 0.01; ***, *p* < 0.001; ns, no significance.

The effects of 5-HT and LL-37 on THP-1-derived macrophages were investigated, with the results shown in Figure 1. Figure 1A–D display the effects of 5-HT and LL-37 on the expressions of inflammatory cytokines, as well as the effects when they were in a mixture. The data showed that LL-37 groups significantly upregulated the expressions of IL-1β, IL-8, and TNF-α in THP-1-derived macrophages compared with the control group. However, after stimulation by LL-37 alone, no obvious increase in VEGF was observed compared with the control group. Nevertheless, in the L5 (LL-37 + 5-HT) group, the levels of four cytokines (IL-1β, IL-8, TNF-α, and VEGF) in the L5 groups were substantially elevated in comparison to the LL-37 group. Furthermore, whether 5-HT was involved in polarization of THP-1-derived macrophages was investigated by morphological evaluation and flow cytometry analysis. The morphological results showed that, after treatment with PMA, the cells became adherent, bulkier, and more cytoplasmic; however, its morphology did not show any apparent change after THP-1-derived macrophages were treated with LL-37 or 5-HT (Supplementary Information Figures S1 and S2). Meanwhile, the flow cytometry data showed that, in CD11b + THP-1 cells, a remarkable increase was observed, ranging from 9.87 ± 4.03% to 70.6 ± 2.49% subsequent to treatment with PMA (Figure 1E,G). As shown in Figure 1F, a significant increase in the M1 macrophages (CD86+/CD206−) was observed after treatment with LL-37, reaching 16.75 ± 0.55%. In comparison, the proportion of M1 macrophages in the control group was 10.85 ± 0.75%, and the groups treated with 5-HT alone remained 10.55 ± 0.05%. However, after treatment with L5, the proportion of M1 macrophages further significantly increased to 30.73 ± 0.43%. Nevertheless, in the M2 macrophages (CD86−/CD206+), no difference was found among all groups (Figure 1F,H).

### 2.2. Effects of 5-HT and LL-37 on HTR mRNA Expressions and Localizations of HTR3A/5-HT

**Figure 2:**
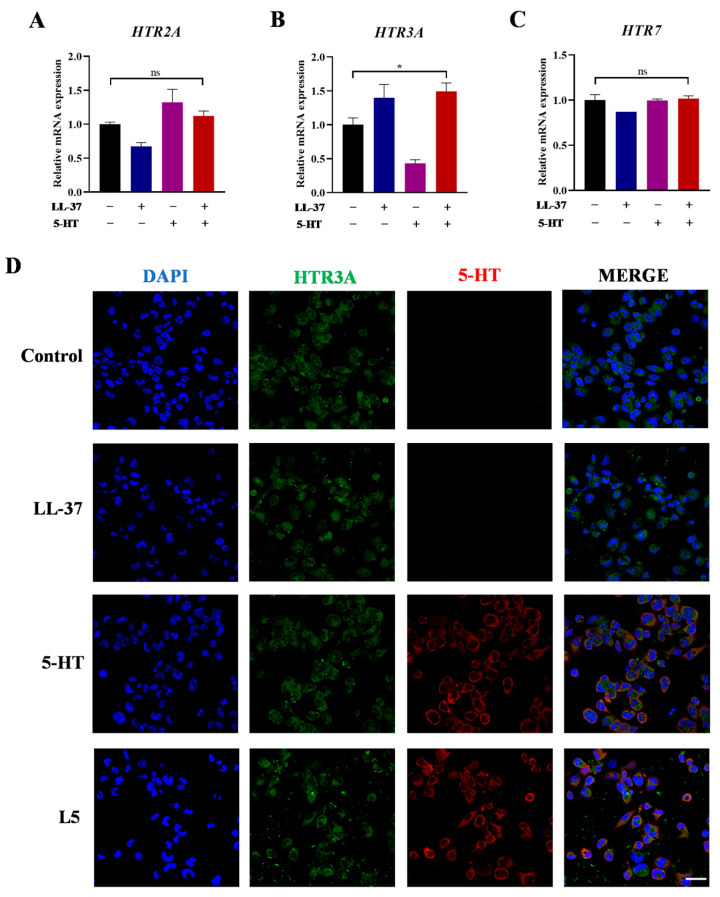
Effects of 5-HT and LL-37 on *HTR* mRNA expressions and localization of HTR3A/5-HT in THP-1-derived macrophages. (**A**–**C**) After incubation with LL-37 or 5-HT for 24 h, the qPCR analysis of the mRNA expressions of *HTR2*, *HTR3A*, and *HTR7* of control (black), LL-37 (blue), 5-HT (purple), and L5 (red) treated cell groups. (**D**) The immunofluorescence staining for HTR3A (green) and 5-HT (red) in THP-1-derived macrophages. Scale bar 25 µm. Data represent mean ± SEM for three independent experiments. *, *p* < 0.05; ns, no significance.

It has been reported that the mRNAs of *HTR2*, *HTR3A*, and *HTR7* showed the top three highest expression abundances in THP-1 cells; thus, they are key receptors for the 5-HT signaling pathway [17]. To explore the mechanism that 5-HT further exacerbated the LL-37-induced inflammatory response, the mRNA expressions of these three receptors were evaluated on THP-1-derived macrophages. The analysis demonstrated that L5 group only triggered a significant elevation in the mRNA expression of *HTR3A* (*, *p* < 0.05), while *HTR2* and *HTR7* exhibited no significant variance (Figure 2A–C). Moreover, to illustrate whether there is an interaction between 5-HT and HTR3A, both 5-HT and HTR3A were labeled to indicate their localization in THP-1-derived macrophages. The immunofluorescent results demonstrated that the subcellular localization of HTR3A was in the cytoplasm of THP-1-derived macrophages. After exposure to 5-HT, it was also in the cytoplasm and was colocalized with HTR3A (Figure 2D).

### 2.3. Roles of HTR3A in L5-Induced THP-1-Derived Macrophages

**Figure 3:**
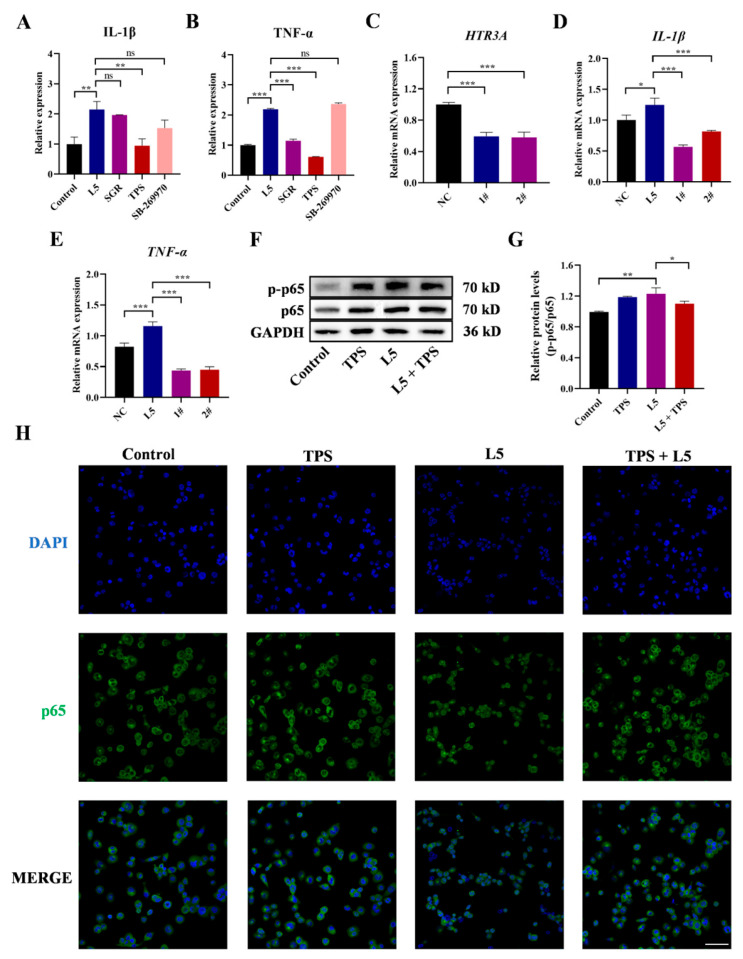
Roles of HTR3A in L5-induced THP-1-derived macrophages. (**A**,**B**) After treatment with SGR, TPS or SB-269970 for 1 h and stimulation by L5 for 24 h, the ELISA results of the expressions of IL-1β and TNF-α of control (black), L5 (blue), L5 + SGR (purple), L5 + TPS (red), and L5 + SB-269970 (pink) treated cell groups. (**C**) After treatment for 24 h, the qPCR analysis of mRNA expressions of *HTR3A* of siNC (black), siHTR3A#1 (blue), and siHTR3A#2 treated cell groups. (**D**,**E**) After stimulation by L5 for 24 h in transfected cells, the qPCR analysis of mRNA expressions of *IL-1β* and *TNF-α* of siNC (black), siNC + L5 (blue), si*HTR3A*#1 + L5 (purple), and si*HTR3A*#2 + L5 (red) treated cell groups. (**F**) After treatment with TPS for 1 h and stimulation by L5 for 1 h, the expressions of p-p65 in THP-1-derived macrophages were tested by Western blot analysis. (**G**) The relative protein levels of p-p65/p65 of control (black), TPS (blue), L5 (purple), and L5 + TPS (red) treated cell groups in Western blot analysis. (**H**) The immunofluorescence analysis of p65 after incubation with TPS for 1 h and stimulation by L5 for 4 h. Scale bar 50 μm. Data represent mean ± SEM for three independent experiments. *, *p* < 0.05; **, *p* < 0.01; ***, *p* < 0.001; ns, no significance.

Subsequently, the selective antagonists corresponding to the three receptors (HTR2: SGR, HTR3A: TPS, and HTR7: SB-269970) were used to investigate the role of 5-HT receptors in enhanced inflammation induced by L5. The appropriate concentration was selected by the cell viability assay to avoid the influence of concentration on subsequent experiments (Supplementary Information Figure S3). The analysis revealed that, when the HTR3A was blocked by TPS, it impaired the production of IL-1β and TNF-α more severely than when HTR2 or HTR7 was blocked (Figure 3A,B). To further verify whether HTR3A is the mediator of inflammation induced by L5, the effect of L5 on cells with lower expression of *HTR3A* was explored using gene silencing. Gene silencing mediated by two different siRNAs (1# and 2#) triggered a significant decrease (***, *p* < 0.001) in the mRNA expression of *HTR3A* along with *IL-1β* and *TNF-α* compared with the control group (Figure 3C–E). After that, the effects of L5 and TPS on NF-κB were investigated by Western blot and immunofluorescent staining. As is illustrated in Figure 3F–H, L5 treatment promoted the phosphorylation and nuclear translocation of p65 in THP-1-derived macrophages in comparison with the control groups; however, the addition of TPS completely reversed the phosphorylation and nuclear translocation of p65 induced by L5 in THP-1-derived macrophages.

### 2.4. Effects of 5-HT on LL-37-Induced Rosacea-like Mice

**Figure 4:**
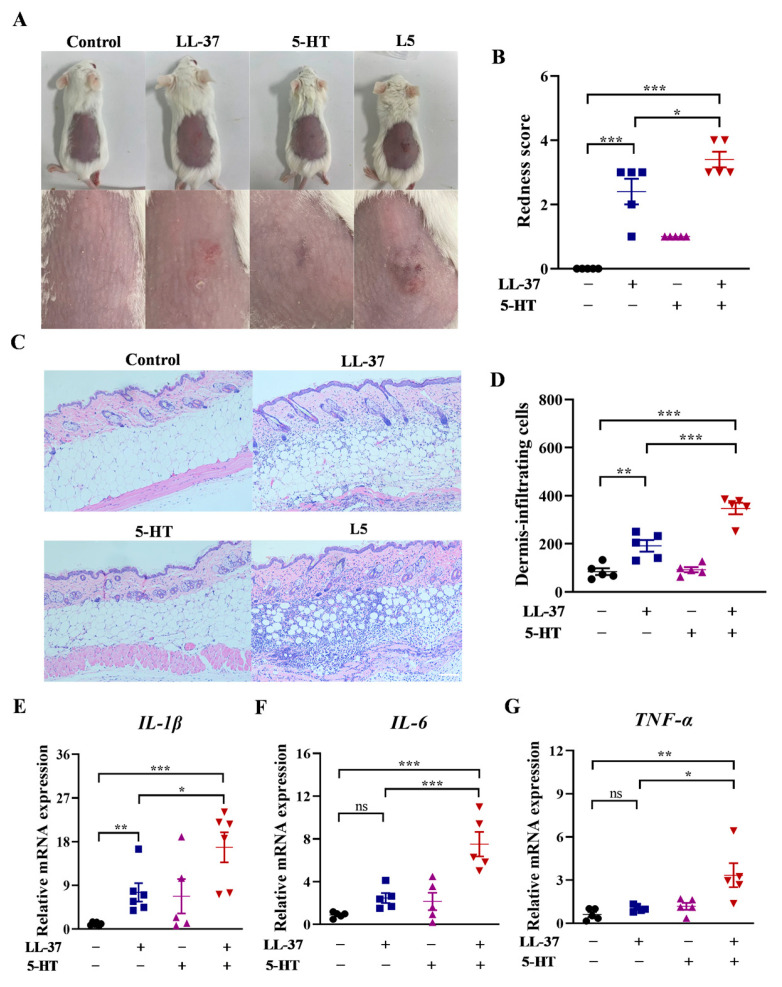
Effects of 5-HT on LL-37-induced rosacea-like mice. (**A**) The presentation of the dorsal skin of control, LL-37, 5-HT, and L5 treated mouse groups. The below images are enlarged images of the erythema site in the above images. (**B**) The redness score of erythema of control (black), LL-37 (blue), 5-HT (purple), and L5 (red) treated mouse groups in the presentation. (**C**) Representative HE staining of skin lesion of control, LL-37, 5-HT, and L5 treated mouse groups. Scale bar 250 μm. (**D**) Quantitative results of infiltrating cells in dermis of control (black), LL-37 (blue), 5-HT (purple), and L5 (red) treated mouse groups in HE staining. (**E**–**G**) The mRNA expressions of *IL-1β*, *IL-6*, and *TNF-α* in skin lesions of control (black), LL-37 (blue), 5-HT (purple), and L5 (red) treated mouse groups. Data represent the mean ± SEM for five independent experiments. *, *p* < 0.05; **, *p* < 0.01; ***, *p* < 0.001; ns, no significance.

To investigate whether 5-HT has proinflammatory effects on mice, we analyzed whether there are changes in the concentration of 5-HT between rosacea-like mice and control mice. Analysis revealed the concentration of 5-HT along with the mRNA expression of *TPH1* (the key enzyme of 5-HT synthesis) was significantly elevated in rosacea-like mice (Supplementary Information Figure S4). Then, the 5-HT was injected intradermally to observe its effects on phenotype of rosacea. In the LL-37 group, apparent erythema of rosacea emerged and, in the 5-HT group, no erythema was induced. However, in the L5 group, the rosacea-like phenotypes induced by LL-37 were further deteriorated by 5-HT (Figure 4A). Meanwhile, 5-HT further increased the redness scores of LL-37-induced rosacea-like mice, although the redness scores in the group treated with 5-HT alone exhibited no variance with the control group (Figure 4B). Histological analysis showed that the infiltration cells and the skin thickness were also further increased after intradermal injection of L5, compared with LL-37-induced rosacea-like mice. However, the results exhibited no significant variance for the number of infiltration cells and the skin thickness between 5-HT and control groups (Figure 4C,D). The mRNA expressions of *IL-1β*, *IL-6*, and *TNF-α* in pathological skin lesions were further increased through intradermal injection of L5 compared with LL-37 treatment, while these mRNA expression levels in 5-HT groups had no significant difference compared with control groups (Figure 4E–G).

### 2.5. Effects of HTR3A Antagonist TPS on L5-Induced Rosacea-like Mice

**Figure 5:**
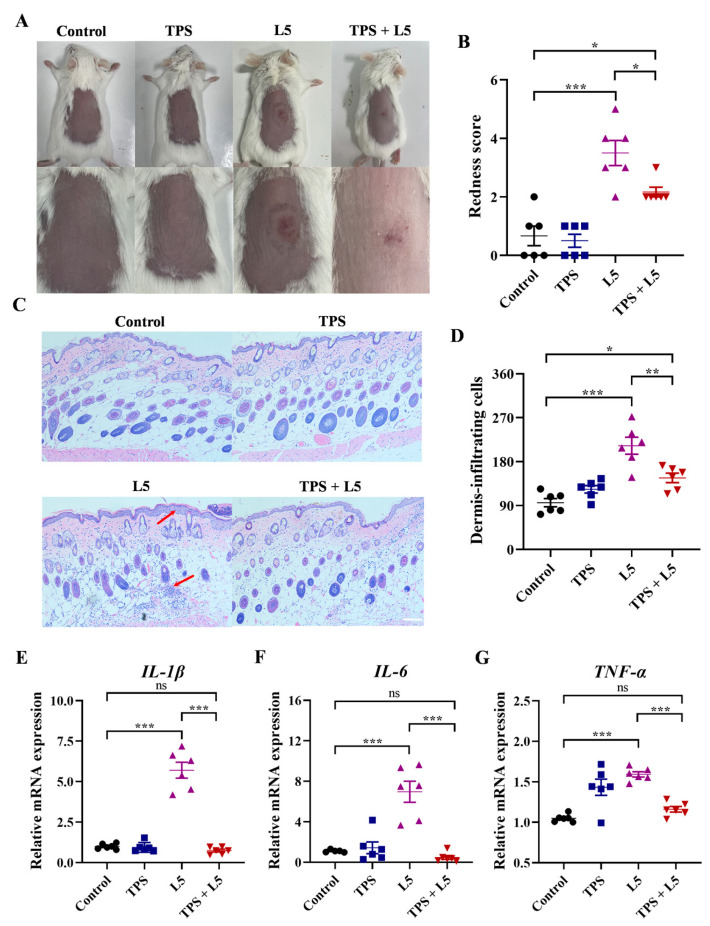
Effects of HTR3A antagonist TPS on L5-induced rosacea-like mice. (**A**) The presentation of the dorsal skin of control, TPS, L5, and TPS + L5 treated mouse groups. The below images are enlarged images of the erythema site in the above images. (**B**) The redness score of erythema of control (black), TPS (blue), L5 (purple), and TPS + L5 (red) treated mouse groups in the presentation. (**C**) Representative HE staining of skin lesions of control, TPS, L5, and TPS + L5 treated mouse groups. The differences between L5 and control group are highlighted by red arrows. Scale bar 250 µm. (**D**) Quantitative results of infiltrating cells in dermis in HE staining. (**E**–**G**) The mRNA expressions of *IL-1β*, *IL-6*, and *TNF-α* in skin lesions of control (black), TPS (blue), L5 (purple), and TPS + L5 (red) treated mouse groups. Data represent mean ± SEM for six independent experiments. *, *p* < 0.05; **, *p* < 0.01; ***, *p* < 0.001.

To conduct a more in-depth exploration of the function of HTR3A in dermatitis of L5-induced rosacea-like mice, the TPS as a selective antagonist of HTR3A was evaluated. The macroscopic images showed that the backs of mice had different appearances between LL-37 and control groups (Figure 5A). The redness score further demonstrated that the HTR3A antagonist TPS significantly inhibited the L5-induced rosacea-like dermatitis, while TPS itself did not cause any changes in skin manifestations (Figure 5B). Meanwhile, the histological analysis demonstrated that the infiltration of inflammatory cells and skin thickness induced by L5 was completely reversed after TPS treatment (Figure 5C,D). Furthermore, the increased mRNA expressions of *TNF-α*, *IL-1β*, and *IL-6* induced by L5 were significantly relieved after TPS treatment in mouse lesions (Figure 5E–G).

## 3. Discussion

In previous research regarding rosacea, keratinocytes have attracted much attention from numerous researchers because of its significance in skin structure and function [18]. However, macrophages are a crucial constituent of the immune defense framework in skin; few studies have comprehensively and systematically elucidated the specific mechanism through which they impact rosacea [19,20]. Furthermore, neurotransmitter 5-HT has been found to be able to regulate the innate immune function related to macrophages [21]. Although 5-HT has been found to modulate inflammation-related diseases, most of them are mainly focused on the intestine and neurons, and the underlying mechanism of 5-HT affecting rosacea has not been reported [12,13,17].

THP-1 cells can be differentiated into macrophages, and these cells play a crucial role in cutaneous inflammatory responses. When studying cutaneous inflammatory diseases, THP-1 cells can be used to mimic the in vivo inflammatory environment to investigate the release of inflammatory factors and the activation mechanisms of immune cells [22]. Therefore, in this study, a rosacea-like model using THP-1 cells was designed to explore the impact of 5-HT on it. LL-37, a cationic antimicrobial peptide, whose expression is increased in the central face of patients and can trigger an innate immune response to promote inflammation [23]. Currently, a few studies have elucidated the research related to rosacea stimulated by LL-37 in THP-1-derived macrophages [24]. Hence, to explicate the intricate correlation between 5-HT and rosacea, we carried out an in-depth evaluation of the impacts of 5-HT on rosacea-like inflammation within THP-1-derived macrophages.

In the cytokine secretion assays, the LL-37 alone upregulated IL-1β, IL-8, and TNF-α, indicating that THP-1-derived macrophages could be stimulated to develop inflammation by LL-37 [25]. But LL-37 failed to induce VEGF elevation. According to the reports by Reynoso-Roldán et al., LL-37 regulated the expression of VEGF in a time-dependent manner. After stimulation by LL-37 for 24 h, the expression of VGEF increased slightly, being half of that observed after 48 h [26]. In our research, there was no difference between the control and LL-37 group. It is likely that this is due to insufficient stimulation time. After cells were incubated with L5, the expressions of IL-1β, IL-8, and TNF-α were significantly increased compared with incubation with LL-37 alone, suggesting that there is a synergistic effect between LL-37 and 5-HT to exacerbate inflammation. For VEGF, after incubation with L5, its expression was significantly increased compared with incubation with 5-HT alone. It is widely known that 5-HT has angiogenic effects; therefore, it is quite understandable that 5-HT upregulated the VEGF expression [27]. Due to the synergistic effect between LL-37 and 5-HT, VEGF expression was further increased. The results of macrophage polarization analysis indicated that PMA pretreatment induced macrophage-like changes. Meanwhile, LL-37 increased the precent of M1 macrophages (CD86+/CD206−), and this was further significantly enhanced through the combined use of LL-37 and 5-HT, while the percent of M2 macrophages remained unchanged. Zhang et al. demonstrated that LL-37 not only impaired M2 polarization but also promoted the phenotypic skew from M2 to M1 macrophages [28]. This mechanism may explain why the combined stimulation with LL-37 and 5-HT failed to induce a more diverse cellular response, consequently resulting in no significant changes in the proportion of M2 macrophages.

The 5-HT receptor is the primary pathway for cellular uptake of 5-HT, and previous research has indicated that receptor activation is key to inducing phenotypic alterations in macrophages [29,30,31]. Evaluation of the top three mRNA expressions of *HTR2*, *HTR3A*, and *HTR7* revealed that exposure to L5 only triggered a significant elevation of the mRNA expression of *HTR3A*. This implies that HTR3A is likely to have a substantial function in regulating the inflammatory response. The absence of significant differences in *HTR2* and *HTR7* mRNA expressions indicates that their roles may be less prominent or involve different regulatory mechanisms. The immunofluorescence staining results showed the cytoplasmic subcellular localization of HTR3A and its colocalization with 5-HT with HTR3A in THP-1-derived macrophages. It implied that the interaction between 5-HT and HTR3A occurs in the cytoplasmic compartment, which could potentially affect intracellular signaling pathways related to rosacea-like inflammation.

The use of selective antagonists provided crucial insights into the role of specific 5-HT receptors in L5-induced inflammation. The finding demonstrated that blocking HTR3A with TPS had the most significant impact on downregulating IL-1β and TNF-α production, indicating that HTR3A may be a key mediator in the enhanced inflammatory effect of L5. In further research, we also found that gene silencing of *HTR3A* using siRNAs triggered a significant reduction in the mRNA expression of *HTR3A* and the secretions of *IL-1β* and *TNF-α*, confirming the direct role of HTR3A in regulating the inflammatory response. The NF-κB serves as a crucial regulator in inflammatory diseases, including rosacea [32,33]. Considering the impact of the association on macrophage functions, this study also investigated NF-κB simultaneously. As a result, it was found that the promotion of phosphorylation and nuclear translocation of p65 was observed in the L5 group, and TPS reversed this process, which suggests that the NF-κB and HTR3A-mediated inflammatory pathway may be involved in this process. But, interestingly, in the treatment with TPS alone, the results of Western bolt showed that it promoted the expression of p65. For this phenomenon, it is suspected that the concentration of TPS played a major role. As reported by Reem et al., at low concentrations, TPS effectively inhibited the expression of NF-κB. However, as the TPS concentration increased, this inhibitory effect diminished [34]. We postulated the existence of a negative feedback regulation mechanism, indicating that the concentration of TPS did not exhibit a straightforward positive correlation with its inhibitory effect on NF-κB expression.

As a second messenger in cells, calcium ions regulate various signaling and physiological functions, including inflammatory response [35,36]. This study revealed that LL-37 triggered rosacea-like inflammation in THP-1-derived macrophages. However, after exposure to high concentration of 5-HT, the ion channel HTR3A was overactivated. Consequently, this over-activation led to a substantial influx of calcium ions [37]. The resulting imbalance in intracellular calcium concentration is presumed to be the key factor contributing to the amplification of the LL-37-induced rosacea-like inflammation.

In vitro study showed that 5-HT had a promoting effect on the LL-37-induced inflammation in THP-1-derived macrophages. However, there are many cell populations recruited by the releasing of neurotransmitters, which are also critical in rosacea pathogenesis. Neutrophils was reported to be associated with the severity of rosacea [38], while 5-HT was demonstrated to mediate neutrophil recruitment through its metabolites [39]. Thus, it can be observed that neutrophils may also play a crucial role in the 5-HT-mdiated rosacea inflammation mediated. Zhang et al. reported that capsaicin, which also serves as a neurotransmitter like 5-HT, exacerbates rosacea-like inflammation by activating γδ T cells [7]. Additionally, the dysregulation of other types of T cells which express high levels of HTRs can also promote the development of rosacea inflammation [8,40]. Further study in these cell populations is warranted to elucidate the underlying mechanisms of 5-HT-mediated neurogenic inflammation in rosacea.

In vivo analysis demonstrated that the levels of 5-HT and *TPH1* mRNA expression were elevated in rosacea-like mice, implying that LL-37 promoted biosynthesis and secretion of 5-HT, which could lead to a bad inflammatory feedback loop. The exacerbation of rosacea phenotypes, including erythema, telangiectasia, and increased redness scores, further supported its proinflammatory role by 5-HT injection. The histological and cytokine expression changes also confirm the exacerbating effect of 5-HT on LL-37-induced inflammation, while 5-HT alone had minimal impact on control mice. These data were consistent with the cell cytokines assays in this study. Additionally, the HTR3A antagonist TPS significantly inhibited rosacea-like symptoms, reversed histological changes, and reduced the mRNA expressions of cytokines in rosacea-like mice models. The TPS reversed the pathological phenotype induced by L5 in rosacea-like mice, thereby presenting a valuable option for the clinical treatment of rosacea. It is highly plausible that this reversal was because the antagonism against HTR3A blocked NF-κB signaling, which was activated by 5-HT. Nevertheless, there exist other potentialities as well, as research has investigated that acute and chronic itch could be induced by 5-HT; also, through HTR3A [41], 5-HT may activate NF-κB by triggering the specific itch-related intracellular mechanism after binding to HTR3A.

This research revealed that TPS alleviated the L5-induced rosacea-like inflammation, suggesting its potential as an immune suppressant. While TPS is traditionally classified as an antiemetic targeting HTR3A, other emerging evidence also highlights its broader immunomodulatory roles. For instance, Li et al. reported that treatment with TPS suppressed colorectal tumorigenesis in an azoxymethane/dextran sodium sulfate (AOM/DSS)-induced murine model of colorectal cancer [17]. Liu et al. demonstrated that TPS inhibits sepsis by repressing hyper-inflammation in a rat model [42]. These properties align with our results, supporting TPS’s utility as a novel immune-suppressive agent in inflammatory conditions like rosacea. Its immunosuppressive properties and potential for autoimmune disease treatment merit further study.

Although TPS has exhibited favorable tolerability in clinical settings, systemic suppression of 5-HT signaling warrants cautious risk–benefit evaluation. Specifically, HTR3A receptor antagonism—the primary mechanism underlying its antiemetic effects—may induce dose-dependent gastrointestinal hypomotility and transient neurological symptoms [43]. Chronic 5-HT pathway modulation could theoretically impair serotonergic neurotransmission and platelet activation [44]. Future studies should prioritize systematic evaluations of chronic toxicity in preclinical models, with particular emphasis on potential neurological adverse effects.

## 4. Materials and Methods

### 4.1. Cell Culture and Treatments

THP-1 monocytes (SCSP-567) were obtained from Cell Bank/Stem Cell Bank, Chinese Academy of Sciences, and cultured in RPMI 1640 medium (Gibco, Waltham, MA, USA) containing 10% fetal bovine serum (New Zerum, Christchurch, New Zealand) and 1% antibiotics (Gibco, USA). THP-1 monocytes were inoculated into 60 mm culture dishes at a density of 6 × 10^5^ cells/mL. At the same time, phorbol-12-myristate-13-acetate (PMA; Sigma Aldrich, St. Louis, MO, USA) with a final concentration of 100 ng/mL was added into the dish and incubated for 24 h to induce them to differentiate into macrophages. Before any treatments, cells were rested in RPMI 1640 for 24 h [45].

Two modified siRNAs of *HTR3A* and the siRNA of the negative control were synthesized by Tsingke Biotechnology (Beijing, China). Lipofectamine 8000 reagent (Beyotime, Beijing, China) was used to carry out the transfection of siRNA into THP-1-derived macrophages. The cells were employed for the experiments only after being transfected for 24 h. The sequences of siRNA are listed in Supplementary Information Table S1.

### 4.2. Animal Experiments

BALB/c mice (6–8 weeks old; female) were purchased from Sbf (Suzhou, China) Biotechnology Company with the license number SCXK (Suzhou, China) 2022-0006. All mice were housed in the Laboratory Animal Center of Jiangsu Institute of Parasitic Diseases, which was accredited with the license number SYXK (Jiangsu, China) 2022-0048. Animal experiments conducted were in accordance with the guidelines of the Institutional Animal Care and Use Committee (IACUC) of China and approved by the Laboratory Animal Ethics Committee of Jiangsu Institute of Parasitic Diseases (License number: JIPD-IACUC-2024121).

After the adaptation period, mouse backs were shaved 24 h before injection. LL-37 (Selleck, Houston, TX, USA) was injected intradermally at a concentration of 320 μM in a total volume of 40 μL per injection, with treatments delivered every 12 h for a total of 4 administrations. 5-HT (Sigma Aldrich, St. Louis, MO, USA) was injected intradermally at a dose of 25 μg per 40 μL volume, administered once daily for 3 consecutive days starting 24 h before the first LL-37 injection [46]. Tropisetron (TPS; Bide Pharmatech, Shanghai, China) was administered intraperitoneally at a dose of 20 mg/kg body weight per injection, delivered once daily for 5 consecutive days starting 48 h before the initial 5-HT injection [47]. The photos of erythema induced by LL-37 and 5-HT injection were taken 12 h after the last injection of LL-37 [48]. The redness score of dorsal skin dermatitis was evaluated by its severity and area on the scale from 0 to 5, 0 indicating no erythema and 5 indicating the most severe erythematous symptom. The serum was collected from the orbit and tested for ELISA kits. The lesional skin tissues were biopsied immediately for RNA extraction and histological analysis.

### 4.3. Histological Analysis

The 4% paraformaldehyde was employed to fix the skin tissues. Next, paraffin was employed for tissue embedding. Thereafter, 5 μm sections were made and stained with hematoxylin and eosin.

### 4.4. Immunofluorescence Analysis

The 4% paraformaldehyde was employed to fix the cell samples for 15 min. The 5% BSA (Beyotime, China) was employed to block for 1 h. Then, anti-5-HT, anti-HTR3A, and anti-p65 antibodies (Invitrogen, Waltham, MA, USA) were incubated overnight at 4 °C. Next day, Alexa Fluor 488-conjugated and Cy3-conjugated (Beyotime, China) secondary antibody were incubated for 1 h, and 4′,6-diamidino-2-phenylindole (DAPI; Invitrogen, USA) was employed to counterstain for 10 min.

### 4.5. Quantitative Real-Time PCR (qPCR) Analysis

Trizol (Beyotime, China) was used to extract the RNA of skin tissues and cells. RNA was reverse-transcribed and qPCR detection was carried out with SYBR Green (Vazyme, Nanjing, China) using a Light Cycler 96 (Roche, Basel, Switzerland) thermal cycler. Relative quantification of gene expression was quantified by ΔCT method, with β-actin as normalization. The sequences of primers are presented in Supplementary Information Table S2.

### 4.6. Flow Cytometry Detection

The cells were adjusted to a number of 5 × 10^6^ per sample. CD16/32 antibody (Invitrogen, USA) was used to block nonspecific FC-mediated interactions. Subsequently, the cells were incubated with CD11b, CD86, and CD206 antibody (Invitrogen, USA) on ice for 40 min. After washing with PBS, cells were resuspended prior to performing the flow cytometry analysis (BD FACSAria III, Franklin Lakes, NJ, USA).

### 4.7. Western Blot Analysis

The collected cell samples were lysed in RIPA (Beyotime, China) with protease and phosphatase inhibitors (MedChemexpress, Shanghai, China). The levels of expression of proteins were quantified by a bicinchoninic acid protein assay kit (Beyotime, China). The prepared protein samples were electrophoresed and transferred to PVDF. PVDF was then blocked with 5% BSA and incubated with primary antibodies overnight. The bands were made visible with the utilization of the HRP substrate. The used primary antibodies were listed as follows: anti-p65 (Beyotime, China), anti-p-p65 (ser563, Beyotime, China), anti-β-actin (Beyotime, China), anti-β-tubulin (Proteintech, Wuhan, China), and anti-GAPDH (Proteintech, China). Images were trimmed for presentation.

### 4.8. Enzyme-Linked Immunosorbent Assay (ELISA)

The collected supernatants of cell samples were centrifuged for ELISA. ELISA kits for human interleukin 1β (IL-1β), interleukin 8 (IL-8), tumor necrosis factor α (TNF-α), vascular endothelial growth factor (VEGF), and mouse 5-HT were obtained from Signalway Antibody (College Park, MD, USA) and performed as per the manufacturer’s instructions.

### 4.9. Statistical Analysis

GraphPad 9.0 was employed to carry out the statistical analysis. Comparisons between two groups were assessed with the Student’s *t*-test. When there were at least 3 groups, statistical significance (*, *p* < 0.05, **, *p* < 0.01, ***, *p* < 0.001) was assessed using one-way analysis of variance (ANOVA) with the Sidak’s multiple comparisons test.

## 5. Conclusions

In conclusion, this research has demonstrated that the neurotransmitter 5-HT plays a crucial role in further promoting LL-37-induced rosacea-like inflammation. The results are supported by the observations obtained from both in vitro and in vivo experiments. Moreover, TPS, as the selective antagonist of HTR3A, completely reversed the exacerbated L5-induced rosacea-like inflammation in THP-1-derived macrophages and mice, demonstrating that TPS have a beneficial effect on the improvement of rosacea. This study preliminarily revealed that HTR3A is the key mediator of 5-HT promoting rosacea-like inflammation, leading to novel therapeutic perspectives for rosacea.

## Data Availability

The data presented in this study are available on request from the corresponding author.

## Supplementary Materials

The following supporting information can be downloaded at: https://www.mdpi.com/article/10.3390/ijms26073156/s1.

## Abbreviations

5-HT	5-Hydroxytryptamine
HTR2	5-Hydroxytryptamine receptor 2
HTR3A	5-Hydroxytryptamine receptor 3A
HTR7	5-Hydroxytryptamine receptor 7
PMA	Phorbol-12-myristate-13-acetate
L5	LL-37 and 5-hydroxytryptamine
SGR	Sarpogrelate,
TPS	Tropisetron
IL-1β	Interleukin-1β
IL-8	Interleukin-8
TNF-α	Tumor necrosis factor α
VEGF	Vascular endothelial growth factor
NF-κB	Nuclear factor-kappa B
