# Supplementary material for: Neurotransmitter 5-HT Further Promotes LL-37-Induced Rosacea-like Inflammation Through HTR3A
Source: Int J Mol Sci. 2025 Mar 28;26(7):3156. doi: 10.3390/ijms26073156 (PMC11988644; doi:10.3390/ijms26073156)
Supplement: Supplementary file 1 [file ijms-26-03156-s001.zip › Supplementary Information.pdf]

Supplementary Information

Neurotransmitter 5-HT Further Promotes LL-37-induced Rosacea-like Inflammation through HTR3A

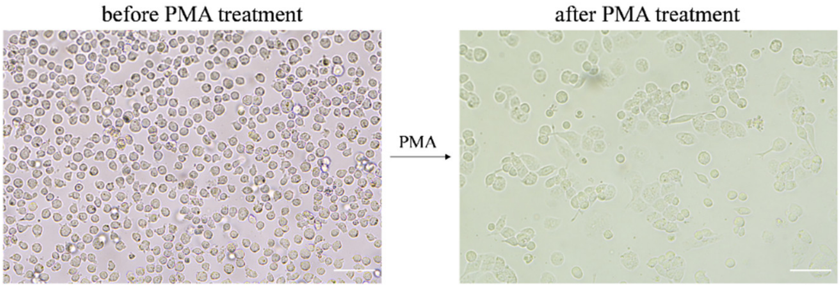

Figure S1. Microscopic photographs of THP-1 cells before and after PMA treatment. Scale bar 50 μm.

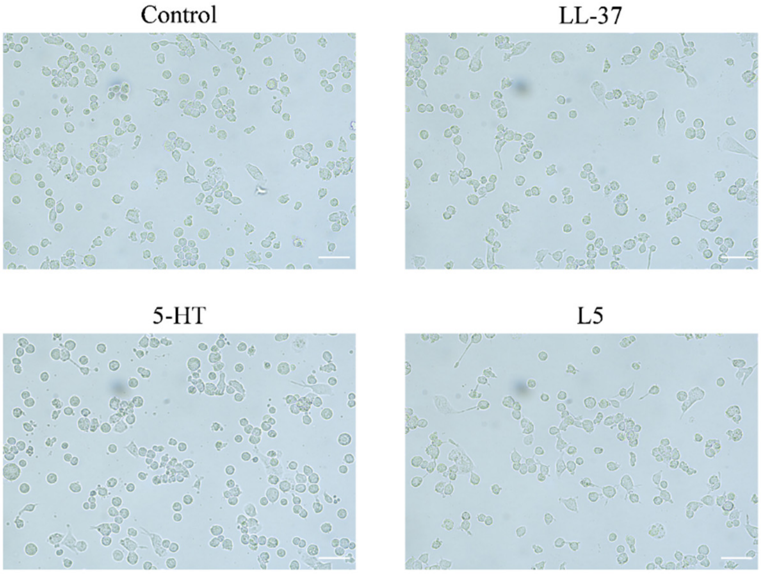

Figure S2. Microscopic photographs of THP-1 derived macrophages after treation with LL-37 or 5-HT for 24 h. Scale bar 50 μm.

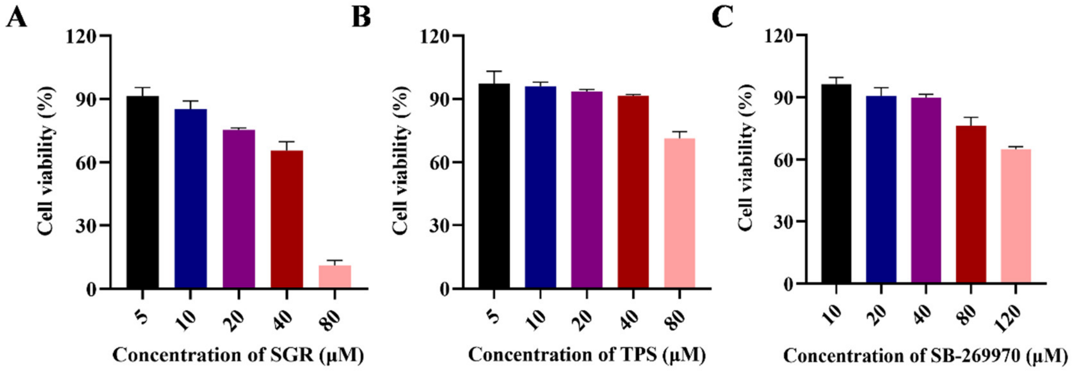

Figure S3. After treatment with different concentrations of SGR, TPS, SB-269970 for 24 h, the cell viability of THP-1 derived macrophages was tested by CCK-8 assays. Data represent mean ± SEM for three independent experiments.

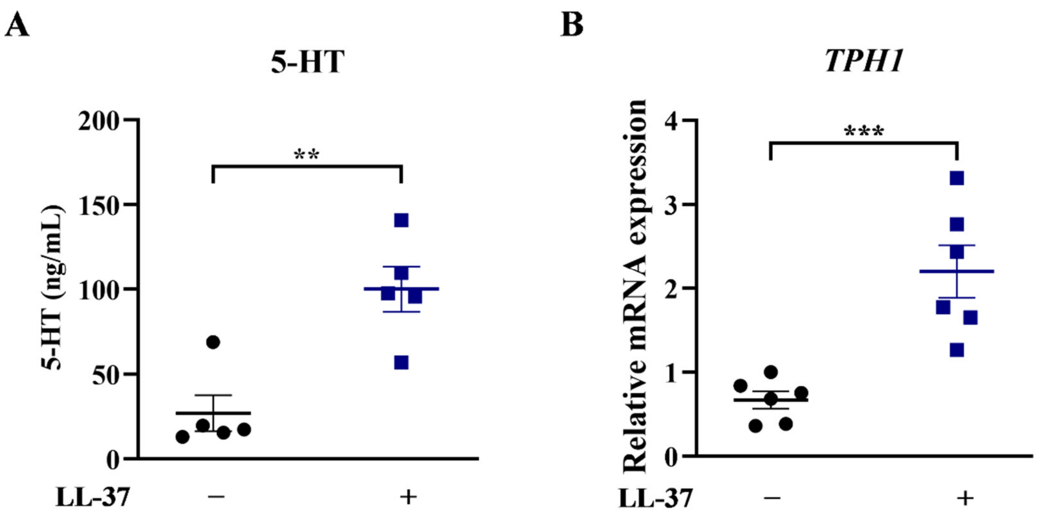

Figure S4. The effects of LL-37 on secretion and biosynthesis of 5-HT in mice. (A) The ELISA results of the concentration of 5-HT in serums of control (black) and LL-37 (blue) treated mouse groups. (B) The qPCR analysis of the mRNA expressions of *THP1* in skin lesions of control (black) and LL-37 (blue) treated mouse groups. Data represent mean ± SEM for at least five independent experiments. **, $p < 0.01$; ***, $p < 0.001$.

Supplementary Tables

Table S1. List of siRNA sequences of negative control (NC) and *HTR3A*

Gene	Strand	Sequence (5'-3')
Human-siNC	Sense	GAGAACUACGAGAGGAUCATT
	Anti-sense	UGAUCCUCUCGUAGUUCUCTT
Human-si*HTR3A*#1	Sense	CAUCGGCACUCCUCUCAUUTT
	Anti-sense	AAUGAGAGGAGUGCCGAUGTT
Human-si*HTR3A*#2	Sense	GUACGUGUAUAUUCGGCAUTT
	Anti-sense	AUGCCGAAUAUACACGUACTT

Table S2. List of primers sequences used for qPCR

Gene	Forward prime (5'-3')	Reverse prime (5'-3')
Human-β-actin	CCCTGGAGAAGAGCTACGAG	CGTACAGGTCTTTGCGGATG
Human-HTR2A	GGAGCCAGGGTCCTACACAG	AGGGCACCACATCACCACAA
Human-HTR3A	TGCCAGAAAAGGTGAAATCC	AGGGCACCACATCACCACAA
Human-HTR7	CCTGTGAGGCAGAATGGGAA	ATGACGGACATGGGGATATA
Human-TNF-α	CCCTGGTATGAGCCCATCTATC	GGCAATGATCCCAAAGTAGACC
Human-IL-1β	CGAATCTCCGACCACCACTAC	AAGGGAAAGAAGGTGCTCAGG
Mouse-GAPDH	AGGTCGGTGTGAACGGATTTG	TGTAGACCATGTAGTTGAGGTCA
Mouse-IL-1β	GATGATAACCTGCTGGTGTGTGAC	GTCGTTGCTTGGTTCTCCTTGTA
Mouse-TNF-α	TGATCCGCGACGTGGAA	ACCGCCTGGAGTTCTGGAA
Mouse-IL-6	GAAATGATGGATGCTACCAAACTG	CTCTGAAGGACTCTGGCTTTGTCT
